# Ethyl 2-(4-chloro­phenyl)-3-(3,5-di­fluoro­phenoxy)acrylate

**DOI:** 10.1107/S1600536808036957

**Published:** 2008-11-20

**Authors:** Hai-Bin Gong, Jie Wang, Ying Liu, Lei Wang

**Affiliations:** a Xuzhou Central Hospital, Xuzhou Cardiovascular Disease Institute, Xuzhou 221009, People’s Republic of China

## Abstract

In the title compound, C_17_H_13_ClF_2_O_3_, a multifunctional aromatic compound, the dihedral angle between the two benzene rings is 51.8 (3)°.

## Related literature

For the biological activities of phenyl­acetate and styrene derivatives, see: Fang *et al.* (2007 ▶); Liu *et al.* (2008 ▶); Shi *et al.* (2007 ▶, 2008 ▶); Zhang, *et al.* (2008 ▶). For bond-length data, see: Allen *et al.* (1987 ▶).
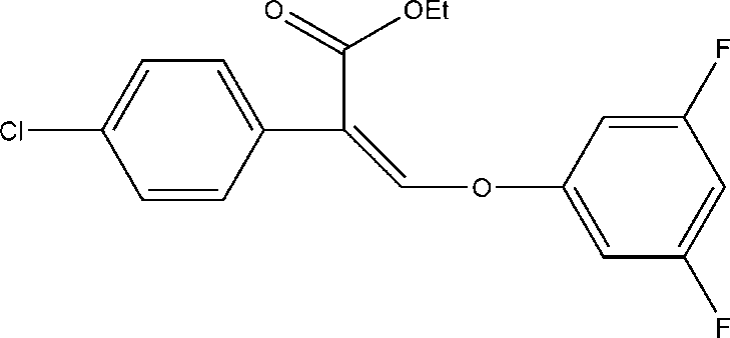


## Experimental

### 

#### Crystal data

C_17_H_13_ClF_2_O_3_
*M* *_r_* = 338.72
Monoclinic, 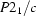

*a* = 9.4999 (17) Å
*b* = 7.6771 (14) Å
*c* = 21.564 (4) Å
β = 91.40 (3)°
*V* = 1572.2 (5) Å^3^
*Z* = 4
Mo *K*α radiation
μ = 0.28 mm^−1^
*T* = 298 (2) K
0.23 × 0.20 × 0.20 mm

#### Data collection

Bruker SMART 1000 CCD area-detector diffractometer
Absorption correction: multi-scan (*SADABS*; Bruker, 2001 ▶) *T*_min_ = 0.939, *T*_max_ = 0.947
3284 measured reflections
3090 independent reflections
2000 reflections with *I* > 2σ(*I*)
*R*_int_ = 0.041

#### Refinement

*R*[*F* ^2^ > 2σ(*F* ^2^)] = 0.071
*wR*(*F* ^2^) = 0.283
*S* = 1.08
3090 reflections
209 parameters
H-atom parameters constrained
Δρ_max_ = 0.47 e Å^−3^
Δρ_min_ = −0.53 e Å^−3^

### 

Data collection: *SMART* (Bruker, 2007 ▶); cell refinement: *SAINT* (Bruker, 2007 ▶); data reduction: *SAINT*; program(s) used to solve structure: *SHELXTL* (Sheldrick, 2008 ▶); program(s) used to refine structure: *SHELXTL*; molecular graphics: *SHELXTL*; software used to prepare material for publication: *SHELXTL*.

## Supplementary Material

Crystal structure: contains datablocks global, I. DOI: 10.1107/S1600536808036957/hg2439sup1.cif

Structure factors: contains datablocks I. DOI: 10.1107/S1600536808036957/hg2439Isup2.hkl

Additional supplementary materials:  crystallographic information; 3D view; checkCIF report

## Figures and Tables

**Fig. 1.**
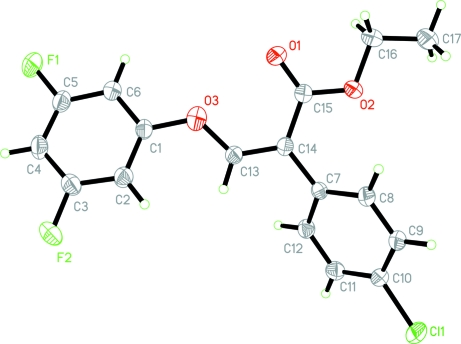
The molecular structure of (I), showing 30% probability displacement ellipsoids and the atom-numbering scheme.

## supplementary crystallographic information

### Comment

Recently, a few phenylacetate and styrene derivatives have been reported with versatile biological activities (Fang *et al.*, 2007; Liu *et al.*, 2008; Shi *et al.*, 2007, 2008; Zhang, *et al.*, 2008). We report herein the title new compound, (I), (Fig. 1).

In compound (I), the dihedral angle between the C1—C6 and C7—C12 phenyl rings is 51.8 (3)°, indicating the molecule is not coplanar. The O3/C13—C15/O1/O2 plane forms dihedral angles of 20.7 (3)° and 47.6 (3)°, respectively, with C1—C6 and C7—C12 phenyl rings. All the bond lengths of the molecule are in normal ranges (Allen *et al.*, 1987).

### Experimental

Equimolar ethyl 3-bromo-2-(4-chlorophenyl)acrylate and 3,5-difluorophenol reacted in chloroform overnight, giving a colorless solution. Block crystals of the compound were formed by gradual evaporation of the solution in air for a week.

### Refinement

H atoms were included in the riding model approximation with C–H = 0.93–0.97 Å and with *U*_iso_(H) = 1.2*U*_eq_(C) and 1.5*U*_eq_(methyl C).

### Crystal data

**Table:**

C_17_H_13_ClF_2_O_3_	*F*_000_ = 696
*M**_r_* = 338.72	*D*_x_ = 1.431 Mg m^−^^3^
Monoclinic, *P*2_1_/*c*	Mo *K*α radiation λ = 0.71073 Å
Hall symbol: -P 2ybc	Cell parameters from 1213 reflections
*a* = 9.4999 (17) Å	θ = 2.5–25.3º
*b* = 7.6771 (14) Å	µ = 0.28 mm^−^^1^
*c* = 21.564 (4) Å	*T* = 298 (2) K
β = 91.40 (3)º	Block, colorless
*V* = 1572.2 (5) Å^3^	0.23 × 0.20 × 0.20 mm
*Z* = 4	

### Data collection

**Table:**

Bruker SMART 1000 CCD area-detector diffractometer	3090 independent reflections
Radiation source: fine-focus sealed tube	2000 reflections with *I* > 2σ(*I*)
Monochromator: graphite	*R*_int_ = 0.041
*T* = 298(2) K	θ_max_ = 26.0º
ω scans	θ_min_ = 1.9º
Absorption correction: multi-scan(SADABS; Bruker, 2001)	*h* = 0→11
*T*_min_ = 0.939, *T*_max_ = 0.947	*k* = 0→9
3284 measured reflections	*l* = −26→26

### Refinement

**Table:**

Refinement on *F*^2^	Secondary atom site location: difference Fourier map
Least-squares matrix: full	Hydrogen site location: inferred from neighbouring sites
*R*[*F*^2^ > 2σ(*F*^2^)] = 0.071	H-atom parameters constrained
*wR*(*F*^2^) = 0.283	*w* = 1/[σ^2^(*F*_o_^2^) + (0.171*P*)^2^ + 0.5784*P*] where *P* = (*F*_o_^2^ + 2*F*_c_^2^)/3
*S* = 1.08	(Δ/σ)_max_ < 0.001
3090 reflections	Δρ_max_ = 0.48 e Å^−^^3^
209 parameters	Δρ_min_ = −0.53 e Å^−^^3^
Primary atom site location: structure-invariant direct methods	Extinction correction: none

### Special details

**Table:**

Geometry. All e.s.d.'s (except the e.s.d. in the dihedral angle between two l.s. planes) are estimated using the full covariance matrix. The cell e.s.d.'s are taken into account individually in the estimation of e.s.d.'s in distances, angles and torsion angles; correlations between e.s.d.'s in cell parameters are only used when they are defined by crystal symmetry. An approximate (isotropic) treatment of cell e.s.d.'s is used for estimating e.s.d.'s involving l.s. planes.
Refinement. Refinement of *F*^2^ against ALL reflections. The weighted *R*-factor *wR* and goodness of fit *S* are based on *F*^2^, conventional *R*-factors *R* are based on *F*, with *F* set to zero for negative *F*^2^. The threshold expression of *F*^2^ > σ(*F*^2^) is used only for calculating *R*-factors(gt) *etc*. and is not relevant to the choice of reflections for refinement. *R*-factors based on *F*^2^ are statistically about twice as large as those based on *F*, and *R*- factors based on ALL data will be even larger.

### Fractional atomic coordinates and isotropic or equivalent isotropic displacement parameters (Å^2^)

**Table:**

	*x*	*y*	*z*	*U*_iso_*/*U*_eq_	
C14	1.0156 (4)	0.6558 (5)	0.20983 (16)	0.0391 (9)	
C7	1.0360 (4)	0.6643 (5)	0.27791 (16)	0.0362 (8)	
C15	1.1233 (4)	0.5884 (5)	0.16872 (17)	0.0428 (9)	
C13	0.8915 (4)	0.7118 (5)	0.18452 (17)	0.0394 (9)	
H13	0.8261	0.7565	0.2117	0.047*	
C8	1.1571 (4)	0.7361 (5)	0.30569 (18)	0.0424 (9)	
H8	1.2298	0.7728	0.2807	0.051*	
C11	0.9438 (4)	0.6248 (6)	0.38096 (19)	0.0509 (10)	
H11	0.8726	0.5864	0.4064	0.061*	
C2	0.6293 (4)	0.8598 (6)	0.13280 (19)	0.0486 (10)	
H2	0.6553	0.9036	0.1716	0.058*	
C1	0.7207 (4)	0.7578 (5)	0.09956 (18)	0.0417 (9)	
C6	0.6816 (5)	0.6998 (6)	0.04035 (19)	0.0510 (11)	
H6	0.7431	0.6344	0.0168	0.061*	
C10	1.0635 (4)	0.6998 (6)	0.40610 (18)	0.0486 (10)	
C12	0.9308 (4)	0.6072 (5)	0.31709 (18)	0.0430 (9)	
H12	0.8500	0.5562	0.3000	0.052*	
C9	1.1716 (4)	0.7540 (5)	0.36912 (19)	0.0470 (10)	
H9	1.2531	0.8020	0.3867	0.056*	
C5	0.5495 (5)	0.7422 (6)	0.0178 (2)	0.0524 (11)	
C4	0.4541 (5)	0.8378 (7)	0.0493 (2)	0.0585 (12)	
H4	0.3650	0.8629	0.0329	0.070*	
C3	0.4983 (4)	0.8946 (6)	0.1066 (2)	0.0542 (11)	
O1	1.1121 (3)	0.5871 (5)	0.11246 (13)	0.0641 (9)	
O2	1.2379 (3)	0.5253 (4)	0.19845 (12)	0.0461 (7)	
O3	0.8532 (4)	0.7092 (5)	0.12313 (16)	0.0664 (9)	
C16	1.3459 (4)	0.4580 (7)	0.1585 (2)	0.0563 (12)	
H16A	1.3703	0.5452	0.1280	0.068*	
H16B	1.3113	0.3555	0.1367	0.068*	
F1	0.5110 (3)	0.6844 (5)	−0.03974 (14)	0.0852 (10)	
C17	1.4696 (5)	0.4135 (9)	0.1967 (2)	0.0833 (18)	
H17A	1.4993	0.5136	0.2202	0.125*	
H17B	1.5443	0.3769	0.1704	0.125*	
H17C	1.4466	0.3206	0.2244	0.125*	
F2	0.4078 (3)	0.9913 (5)	0.14082 (16)	0.0970 (12)	
Cl1	1.07938 (14)	0.7341 (2)	0.48566 (5)	0.0805 (6)	

### Atomic displacement parameters (Å^2^)

**Table:**

	*U*^11^	*U*^22^	*U*^33^	*U*^12^	*U*^13^	*U*^23^
C14	0.0362 (19)	0.043 (2)	0.0375 (19)	0.0008 (16)	−0.0035 (15)	0.0011 (16)
C7	0.0356 (18)	0.0357 (19)	0.0374 (19)	0.0084 (15)	−0.0012 (14)	0.0008 (15)
C15	0.040 (2)	0.048 (2)	0.040 (2)	0.0021 (17)	−0.0007 (16)	0.0006 (17)
C13	0.0382 (19)	0.041 (2)	0.0386 (19)	0.0015 (16)	−0.0023 (15)	−0.0047 (16)
C8	0.0340 (18)	0.050 (2)	0.043 (2)	−0.0018 (17)	−0.0016 (15)	−0.0006 (17)
C11	0.044 (2)	0.065 (3)	0.044 (2)	0.003 (2)	0.0058 (17)	0.005 (2)
C2	0.050 (2)	0.049 (2)	0.047 (2)	0.0039 (19)	−0.0064 (18)	−0.0056 (19)
C1	0.0375 (19)	0.045 (2)	0.042 (2)	0.0017 (17)	−0.0041 (16)	0.0047 (17)
C6	0.050 (2)	0.060 (3)	0.043 (2)	0.010 (2)	−0.0033 (18)	−0.0019 (19)
C10	0.045 (2)	0.065 (3)	0.0356 (19)	0.010 (2)	−0.0018 (16)	−0.0038 (19)
C12	0.0374 (19)	0.047 (2)	0.045 (2)	−0.0024 (17)	−0.0015 (16)	0.0012 (17)
C9	0.038 (2)	0.056 (3)	0.047 (2)	0.0011 (18)	−0.0059 (17)	−0.0103 (19)
C5	0.052 (2)	0.059 (3)	0.045 (2)	0.002 (2)	−0.0103 (19)	−0.001 (2)
C4	0.044 (2)	0.072 (3)	0.059 (3)	0.006 (2)	−0.013 (2)	0.006 (2)
C3	0.048 (2)	0.054 (3)	0.060 (3)	0.011 (2)	−0.0001 (19)	−0.003 (2)
O1	0.0592 (19)	0.095 (3)	0.0375 (16)	0.0233 (18)	0.0006 (13)	0.0027 (16)
O2	0.0393 (14)	0.0551 (17)	0.0442 (15)	0.0111 (13)	0.0049 (11)	−0.0029 (12)
O3	0.060 (2)	0.080 (2)	0.058 (2)	0.0072 (17)	−0.0116 (15)	−0.0001 (17)
C16	0.050 (2)	0.068 (3)	0.051 (2)	0.010 (2)	0.011 (2)	−0.007 (2)
F1	0.076 (2)	0.124 (3)	0.0538 (16)	0.0185 (19)	−0.0268 (14)	−0.0219 (17)
C17	0.058 (3)	0.126 (5)	0.066 (3)	0.038 (3)	0.004 (2)	−0.009 (3)
F2	0.070 (2)	0.134 (3)	0.087 (2)	0.053 (2)	−0.0065 (16)	−0.029 (2)
Cl1	0.0637 (8)	0.1398 (14)	0.0378 (6)	0.0078 (8)	−0.0051 (5)	−0.0114 (7)

### Geometric parameters (Å, °)

**Table:**

C14—C13	1.357 (5)	C6—C5	1.374 (6)
C14—C15	1.465 (5)	C6—H6	0.9300
C14—C7	1.478 (5)	C10—C9	1.380 (6)
C7—C12	1.395 (5)	C10—Cl1	1.739 (4)
C7—C8	1.397 (5)	C12—H12	0.9300
C15—O1	1.215 (5)	C9—H9	0.9300
C15—O2	1.340 (5)	C5—F1	1.359 (5)
C13—O3	1.364 (5)	C5—C4	1.361 (7)
C13—H13	0.9300	C4—C3	1.368 (6)
C8—C9	1.378 (5)	C4—H4	0.9300
C8—H8	0.9300	C3—F2	1.365 (5)
C11—C10	1.374 (6)	O2—C16	1.450 (5)
C11—C12	1.386 (5)	C16—C17	1.459 (6)
C11—H11	0.9300	C16—H16A	0.9700
C2—C3	1.380 (6)	C16—H16B	0.9700
C2—C1	1.382 (6)	C17—H17A	0.9600
C2—H2	0.9300	C17—H17B	0.9600
C1—C6	1.394 (6)	C17—H17C	0.9600
C1—O3	1.397 (5)		
			
C13—C14—C15	118.8 (3)	C11—C12—C7	121.5 (4)
C13—C14—C7	118.5 (3)	C11—C12—H12	119.2
C15—C14—C7	122.7 (3)	C7—C12—H12	119.2
C12—C7—C8	117.3 (3)	C8—C9—C10	119.0 (4)
C12—C7—C14	120.6 (3)	C8—C9—H9	120.5
C8—C7—C14	122.0 (3)	C10—C9—H9	120.5
O1—C15—O2	121.7 (3)	F1—C5—C4	117.6 (4)
O1—C15—C14	124.1 (3)	F1—C5—C6	118.0 (4)
O2—C15—C14	114.2 (3)	C4—C5—C6	124.4 (4)
C14—C13—O3	126.4 (4)	C5—C4—C3	115.5 (4)
C14—C13—H13	116.8	C5—C4—H4	122.3
O3—C13—H13	116.8	C3—C4—H4	122.3
C9—C8—C7	121.8 (4)	F2—C3—C4	118.5 (4)
C9—C8—H8	119.1	F2—C3—C2	117.3 (4)
C7—C8—H8	119.1	C4—C3—C2	124.2 (4)
C10—C11—C12	119.1 (4)	C15—O2—C16	115.0 (3)
C10—C11—H11	120.5	C13—O3—C1	124.4 (3)
C12—C11—H11	120.5	O2—C16—C17	108.7 (4)
C3—C2—C1	118.0 (4)	O2—C16—H16A	110.0
C3—C2—H2	121.0	C17—C16—H16A	110.0
C1—C2—H2	121.0	O2—C16—H16B	110.0
C2—C1—C6	119.9 (4)	C17—C16—H16B	110.0
C2—C1—O3	122.3 (4)	H16A—C16—H16B	108.3
C6—C1—O3	117.7 (4)	C16—C17—H17A	109.5
C5—C6—C1	118.0 (4)	C16—C17—H17B	109.5
C5—C6—H6	121.0	H17A—C17—H17B	109.5
C1—C6—H6	121.0	C16—C17—H17C	109.5
C11—C10—C9	121.3 (4)	H17A—C17—H17C	109.5
C11—C10—Cl1	120.3 (3)	H17B—C17—H17C	109.5
C9—C10—Cl1	118.4 (3)		
			
C13—C14—C7—C12	45.7 (5)	C8—C7—C12—C11	1.6 (6)
C15—C14—C7—C12	−133.9 (4)	C14—C7—C12—C11	−175.6 (4)
C13—C14—C7—C8	−131.3 (4)	C7—C8—C9—C10	−0.1 (6)
C15—C14—C7—C8	49.1 (5)	C11—C10—C9—C8	1.6 (7)
C13—C14—C15—O1	3.9 (6)	Cl1—C10—C9—C8	−176.6 (3)
C7—C14—C15—O1	−176.5 (4)	C1—C6—C5—F1	−179.5 (4)
C13—C14—C15—O2	−175.6 (4)	C1—C6—C5—C4	0.3 (7)
C7—C14—C15—O2	3.9 (6)	F1—C5—C4—C3	−179.3 (4)
C15—C14—C13—O3	1.5 (6)	C6—C5—C4—C3	0.9 (7)
C7—C14—C13—O3	−178.1 (4)	C5—C4—C3—F2	−179.9 (4)
C12—C7—C8—C9	−1.4 (6)	C5—C4—C3—C2	−0.3 (7)
C14—C7—C8—C9	175.7 (4)	C1—C2—C3—F2	178.1 (4)
C3—C2—C1—C6	2.7 (6)	C1—C2—C3—C4	−1.5 (7)
C3—C2—C1—O3	−177.2 (4)	O1—C15—O2—C16	0.5 (6)
C2—C1—C6—C5	−2.2 (7)	C14—C15—O2—C16	−179.9 (4)
O3—C1—C6—C5	177.8 (4)	C14—C13—O3—C1	175.6 (4)
C12—C11—C10—C9	−1.4 (7)	C2—C1—O3—C13	19.2 (6)
C12—C11—C10—Cl1	176.7 (3)	C6—C1—O3—C13	−160.7 (4)
C10—C11—C12—C7	−0.2 (6)	C15—O2—C16—C17	173.4 (4)
